# Wnt signaling in cardiac development and heart diseases

**DOI:** 10.1007/s11626-024-00917-z

**Published:** 2024-05-06

**Authors:** Keita Horitani, Ichiro Shiojima

**Affiliations:** Department of Medicine II, Kansai Medical University, 2-5-1, Shin-Machi, Hirakata, Osaka 573-1010 Japan

**Keywords:** Wnt5a, Heart development, Myocardial infarction, Heart failure

## Abstract

The Wnt signaling pathway is a fundamental cellular communication system with extensive implications in various organs including the heart. In cardiac homeostasis, it governs essential processes like cellular proliferation, differentiation, and apoptosis, ensuring the heart’s structural and functional integrity from embryonic stages and throughout life. Both canonical and non-canonical Wnt signaling pathways play a critical role during embryonic heart development in a region- and stage-specific manner. Canonical Wnt signaling also plays a significant role in heart diseases such as myocardial infarction and heart failure. However, the role of non-canonical Wnt signaling in heart diseases has not been fully elucidated. Wnt5a is a major ligand that activates non-canonical Wnt pathway, and recent studies start to clarify the role of the Wnt5a signaling axis in cardiac health and disease. In this review, we will briefly summarize the previous findings on the role of Wnt signaling pathways in heart development and diseases, and then focus on the role of Wnt5a signaling in heart failure progression. The multifaceted roles of the Wnt signaling pathway highlight its therapeutic potential for various types of heart diseases.

## Introduction

Myocardial infarction (MI) occurs due to blunted coronary artery blood flow, leading to necrotic death of cardiac muscle. Heart failure is a disease state caused by contractile and/or diastolic dysfunction of the heart, and myocardial infarction and left ventricular hypertrophy, the latter caused by increased hemodynamic overload imposed to the heart, are main causes of left ventricular dysfunction. Cardiovascular disease, especially MI and heart failure, continues to be a leading cause of morbidity and mortality worldwide (Roth *et al*. 2020). Moreover, the number of heart failure patients continues to rise (Okura *et al*. 2008; Roger 2021). Despite significant advances in healthcare technology and therapeutic strategies, the burden of cardiovascular disease remains high, emphasizing the need for innovative approaches to diagnosis, prevention, and treatment (Yusuf *et al*. 2004). One such potential avenue is exploring the role of the Wnt signaling pathway, a fundamental and highly conserved pathway involved in numerous biological processes ranging from embryonic development to adult tissue homeostasis (Nusse and Clevers 2017).

Originally identified for its critical role in embryogenesis (Clevers and Nusse 2012), the importance of Wnt signaling pathway extends into adulthood where it continues to regulate processes such as cell proliferation, differentiation, migration, genetic stability, apoptosis, and stem cell renewal (Clevers 2006). Recently, an intriguing connection has emerged between this multifaceted signaling pathway and adult cardiovascular health, particularly in the context of myocardial infarction and heart failure.

The heart, once considered a terminally differentiated organ with little regenerative capacity, has shown some promise of repair and regeneration. This regenerative potential, although limited, has been attributed to several cellular and molecular mechanisms, with the Wnt signaling pathway playing a crucial role (Gessert and Kühl 2010). The involvement of Wnt signaling in adult heart function, maintenance, and response to injury has become increasingly evident in recent years, prompting intensive investigations into its intricate actions.

In the context of myocardial infarction, where there is extensive loss of cardiomyocytes leading to impaired heart function, understanding the precise mechanisms of Wnt signaling could hold the key to innovative therapeutic strategies. This pathway could theoretically be manipulated to enhance the repair of damaged myocardial tissue, reduce post-infarction complications, and improve overall cardiac function (Tao *et al*. 2016).

Although this promising field of research holds significant potential, the interactions of the Wnt signaling pathway within the complex biology of the adult cardiovascular system are not entirely understood (Naito *et al*. 2010). The dualistic role of the Wnt pathway in cardiovascular biology—as a potential promoter of cardiac repair but also implicated in fibrosis and cardiac remodeling post-injury—adds a layer of complexity. Moreover, given the vastness of the Wnt signaling network and its interaction with other pathways, untangling its role in cardiac health and disease represents a substantial but necessary challenge.

In this review, we will discuss the complex role of the Wnt signaling pathway in adult cardiac function and its potential implications for cardiovascular disease therapeutics.

## Wnt signaling in cardiac development and homeostasis

The Wnt signaling pathway plays a pivotal role in various cellular processes ranging from embryonic development to tissue homeostasis in adult organisms (Clevers and Nusse 2012). One of the most intriguing areas of Wnt signaling research is its implication in cardiac development and homeostasis, providing key insights into the molecular mechanisms governing heart formation and function (Paige *et al*. 2015).

### Cardiac development

During embryonic development, the heart is one of the first organs to form, and the Wnt/β-catenin pathway has been shown to play an essential role in the early specification and differentiation of cardiac progenitor cells (Lian *et al*. 2012). The Wnt pathway, primarily through β-catenin-dependent signaling, regulates the mesodermal commitment to the cardiac lineage, thereby determining the first step of heart formation. Consistent with this notion, activation of Wnt/β-catenin signaling in the early stage of embryonic stem cell differentiation promotes cardiogenesis. However, activation of Wnt/β-catenin signaling in the late stage inhibits cardiogenesis, indicating stage-specific and biphasic roles of canonical Wnt signaling in cardiomyocyte differentiation (Naito *et al*. 2006; Ueno *et al*. 2007). Likewise, hyperactivation of Wnt/β-catenin signaling leads to heart defects in frogs, highlighting the importance of a balanced Wnt pathway for proper cardiac morphogenesis (Lavery *et al*. 2008). Wnt signaling is also implicated in left–right axis determination and loss of Wnt3a signaling in the early embryo results in randomization of the initial looping of the heart tube (Nakaya *et al*. 2005).

It was also previously reported that non-canonical Wnt signaling is required for normal heart development: inhibition of *Wnt11* in frogs disrupts heart formation (Pandur *et al*. 2002), and combined deletion of *Wnt5a/Wnt11* genes in mice results in single-chambered heart due to loss of second heart field progenitors (Cohen *et al*. 2012). Thus, both canonical and non-canonical Wnt signaling are required for normal heart development in a stage- and region-specific manner.

### Cardiac homeostasis

Beyond development, Wnt signaling remains crucial for maintaining heart health and function in adulthood. The heart’s capability to adapt and repair, especially after injuries like myocardial infarction, is partly attributed to modulation of Wnt signaling (Aisagbonhi *et al*. 2011). In adult heart tissues, activation of the Wnt/β-catenin pathway has been associated with fibrotic responses and pathological remodeling, suggesting its involvement in heart disease progression (Zelarayán *et al*. 2008). Recent studies also pointed out the potential of targeting Wnt signaling in therapeutic applications. For instance, inhibiting the Wnt pathway has shown promise in reducing fibrosis and improving cardiac function after myocardial injury (Duan *et al*. 2012). On the other hand, activating the Wnt pathway might hold potential for cardiac regenerative therapies, although such interventions need to be approached with caution given the pathway’s multifaceted roles (Bergmann 2010).

## Canonical Wnt signaling in myocardial infarction

**Figure 1.**
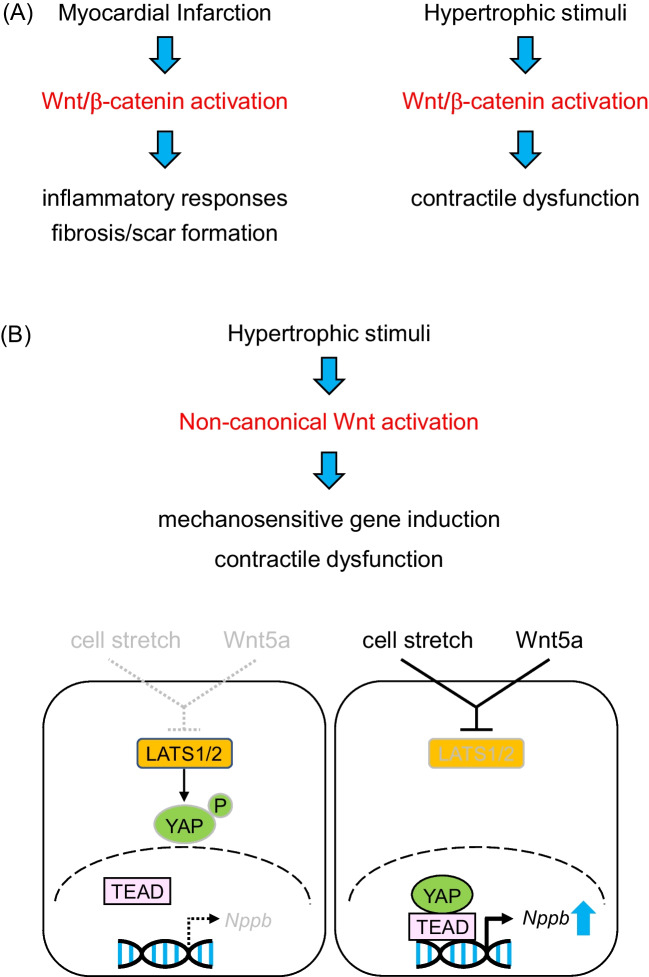
Activation of Wnt signaling contributes to heart failure progression. (***A***)Following myocardial infarction (MI) or in response to hypertrophic stimuli such as pressure overload, canonical Wnt/b-catenin pathway is activated, which promotes inflammatory responses and fibrosis/scar formation in the setting of MI (*left*) and contractile dysfunction in cardiac hypertrophy (*right*). (***B***) In response to hypertrophic stimuli, non-canonical Wnt pathway is also activated, which promotes mechanosensitive gene induction and contractile dysfunction (*upper*). In vitro experiments using cultured cardiac myocytes suggest that YAP is situated downstream of Wnt5a and both cell stretch and Wnt5a are required for mechanosensitive gene induction (*lower*).

Studies have shown that Wnt signaling pathway, particularly the canonical Wnt/β-catenin pathway, is activated following myocardial infarction (Aisagbonhi *et al*. 2011). This activation plays a role in orchestrating the acute inflammatory response, a critical first phase in the healing process post-MI (Frangogiannis 2014). The activated pathway influences the recruitment and activation of immune cells to the infarcted area, including neutrophils and macrophages, which help to clear dead cells and debris (Fig. 1*A*).

Following the initial inflammatory phase, the heart tissue goes through a process of repair characterized by fibrosis, which involves the formation of scar tissue to replace the necrotic myocardium. Activated Wnt signaling promotes the differentiation of cardiac fibroblasts into myofibroblasts (Duan *et al*. 2012), the cells responsible for fibrotic scar formation. These myofibroblasts synthesize extracellular matrix proteins, including collagen, which provides mechanical stability to the damaged heart tissue. However, excessive fibrosis can contribute to heart stiffness and impairment of cardiac function, demonstrating the need for a delicate balance in Wnt signaling activity (Fig. 1*A*).

The pathophysiological role of canonical Wnt signaling in MI was studied using mice with cardiomyocyte-specific β-catenin depletion or β-catenin stabilization in the adult heart (Zelarayán *et al*. 2008). Although β-catenin stabilization in adult cardiac myocytes had no effect on survival after MI, β-catenin depletion in adult cardiac myocytes attenuated left ventricular remodeling and resulted in better survival following MI, supporting the notion that Wnt activation after MI is detrimental for the heart.

## Canonical Wnt signaling in cardiac hypertrophy and heart failure

Canonical Wnt signaling promotes inflammation and fibrosis in the context of cardiac hypertrophy and heart failure. The pathophysiological role of canonical Wnt signaling in this situation was also studied in the same animal models mentioned above (Baurand *et al*. 2007). Although continuous infusion of angiotensin II (AngII) induced cardiac hypertrophy in control mice and in mice with cardiomyocyte-specific β-catenin depletion in the adult heart, AngII infusion in mice with β-catenin stabilization in adult cardiac myocytes resulted in attenuated cardiac hypertrophy and reduced contractile function. This suggests that downregulation of Wnt/β-catenin signaling is required for normal adaptive cardiac hypertrophy and that activation of Wnt/β-catenin signaling is deleterious for the stressed heart (Fig. 1*A*).

## Non-canonical Wnt signaling in cardiac hypertrophy and heart failure

The pathophysiological role of non-canonical Wnt signaling in the adult heart has been elusive. We therefore started to investigate the role of Wnt5a signaling in the adult heart using tamoxifen-inducible *Wnt5a* cardiomyocyte-specific knockout (CKO) mice, which enabled the deletion of *Wnt5a* specifically in adult cardiomyocytes (Kishimoto *et al*. 2023). Following tamoxifen administration, there was a significant reduction in *Wnt5a* expression in the hearts of CKO mice compared to control mice. The mice were then subjected to transverse aortic constriction (TAC), a surgical procedure that induces pressure overload and leads to cardiac hypertrophy and dysfunction (Opie *et al*. 2006). Remarkably, the CKO mice exhibited attenuated chamber dilatation, wall thickness, and contractile dysfunction of the left ventricle post-TAC, compared to control mice. Furthermore, TAC-induced cardiomyocyte hypertrophy and interstitial fibrosis were significantly reduced in CKO hearts, underscoring the cardioprotective effect of *Wnt5a* deletion.

To elucidate the molecular mechanisms underlying these observations, RNAseq analysis was performed on mRNA samples from control and Wnt5a CKO hearts at different time points post-TAC. Differential expression patterns of genes associated with the “cellular response to mechanical stimuli” category were observed, indicating a potential role of Wnt5a in mechanotransduction in the heart (Jaalouk and Lammerding 2009). Subsequent in vitro experiments using neonatal rat ventricular cardiomyocytes (NRVCs) corroborated this observation, demonstrating that Wnt5a is necessary for the upregulation of *Nppb*, a gene associated with cardiac stress, in response to mechanical stretch. Interestingly, treatment with recombinant Wnt5a did not increase *Nppb* expression, suggesting that Wnt5a is necessary but not sufficient for mechanotransduction in cardiac myocytes.

We further investigated the downstream signaling pathways involved in Wnt5a-mediated mechanotransduction and discovered that Wnt5a mediates its effects through the activation of YAP, a transcriptional co-activator implicated in the Hippo signaling pathway (Panciera *et al*. 2017). *Wnt5a* knockdown inhibited the nuclear translocation of YAP induced by cyclic stretch in NRVCs, and *Yap1* knockdown attenuated the stretch-induced upregulation of *Nppb*, confirming the involvement of YAP in mechanotransduction. Pharmacological inhibition of Mst1/2 kinases solidified the conclusion that the Wnt5a-YAP signaling axis mediates mechanotransduction in cardiac myocytes (Fig. 1*B*). This study brings to light the pivotal role of cardiomyocyte-derived Wnt5a in heart failure progression in response to pressure overload. The findings suggest that non-canonical Wnt5a-YAP signaling axis mediates mechanotransduction in cardiac myocytes and contributes to the transition to heart failure. Consistent with this notion, it was previously reported that ROR2 is upregulated in human right ventricle obtained from patients with right ventricular failure, suggesting the involvement of non-canonical Wnt signaling in heart failure progression (Edwards *et al*. 2020). Although the precise mechanism for Wnt5a-mediated cardiac dysfunction remains unclear, it is speculated that activation of mechanosensitive genes contributes to the progression of heart failure following pressure overload (Fig. 1*B*).

Previous studies have investigated the role of Hippo signaling in pressure overload–induced heart failure, yielding conflicting results regarding the cardioprotective or deleterious effects of YAP activation. Cardiac-specific homozygous deletion of YAP results in dilated cardiomyopathy-like phenotype at baseline and heterozygous cardiac-specific YAP knockout mice exhibit exacerbated contractile dysfunction after TAC, suggesting that YAP is cardioprotective (Byun *et al*. 2019). However, YAP activation by cardiac-specific deletion of WW45, a scaffold protein required for LATS1/2 activation by MST1/2, results in enhanced contractile dysfunction after TAC, suggesting that YAP activation in this case is deleterious for the heart (Ikeda *et al*. 2019). Moreover, although both inactivation (heterozygous deletion of YAP) and activation (homozygous deletion of WW45) of YAP promote heart failure in response to pressure overload, combined deletion of YAP (heterozygous) and WW45 (homozygous) in cardiac myocytes is protective against pressure overload–induced cardiac dysfunction (Ikeda *et al*. 2019). These results collectively suggest that YAP must be maintained at appropriate levels for the heart to maintain normal function. It is therefore possible that pressure overload–induced YAP activation is decreased by *Wnt5a* deletion to “appropriate levels for the heart,” leading to maintained contractile function in response to pressure overload.

It should be noted that neutrophil-specific Wnt5a deletion protects the heart from pressure overload–induced dysfunction (Wang *et al*. 2019) and loss of macrophage Wnt secretion improves contractile dysfunction after MI (Palevski *et al*. 2017), indicating a significant role of Wnt ligands derived from both myocytes and non-myocytes in heart failure progression.

These findings not only enhance our understanding of the molecular mechanisms underlying pressure overload–induced cardiac dysfunction but also pave the way for the development of novel therapeutic strategies targeting the Wnt5a-YAP signaling axis in cardiac diseases associated with pressure overload.

## Therapeutic implications

The Wnt signaling pathway, an integral component of cellular communication, holds substantial promise in the realm of cardiovascular therapeutics. Both myocardial infarction and the subsequent progression to heart failure have been closely linked to this pathway, highlighting the potential for innovative therapeutic interventions (Pahnke *et al*. 2016).

### Wnt inhibitors and myocardial infarction

In the wake of MI, the cardiac tissue undergoes significant remodeling. This process often culminates in structural and functional alterations of the heart, which can contribute to long-term morbidity. The activation of the Wnt pathway during this phase offers potential therapeutic avenues. Studies in animal models have illuminated that Wnt pathway modulation, particularly its inhibition, may be pivotal in enhancing cardiac functional recovery and curtailing scar formation.

Several Wnt inhibitors, including secreted frizzled–related proteins (SFRPs) and insulin-like growth factor binding protein-4 (IGFBP-4), have been explored for their therapeutic potential. These inhibitors have shown a capacity to modulate the Wnt pathway and significantly enhance cardiac outcomes post-MI (Mirotsou *et al*. 2007; He *et al*. 2010; Wo *et al*. 2016). This has spurred interest in the pharmaceutical realm, with agents targeting the Wnt pathway being pushed into advanced stages of clinical trials (Zhang *et al*. 2023).

### Wnt signaling in heart failure therapy

The continuum of cardiac diseases often leads from MI to chronic heart failure, a debilitating condition with limited therapeutic options. The Wnt signaling pathway, especially the Wnt5a component, has emerged as a prominent factor in this transition. Experimental evidence suggests that by modulating aberrant Wnt signaling, there is potential to slow or even reverse some aspects of heart failure progression. Animal models have provided a framework, demonstrating reduced cardiac fibrosis and improved function with Wnt pathway inhibition.

### Challenges in targeting Wnt signaling

The therapeutic journey is not devoid of challenges. The Wnt signaling maze, with its myriad of proteins and crosstalk with other pathways, is intricate (MacDonald *et al*. 2009). This complexity poses a dual challenge: ensuring that therapeutic modulation does not inadvertently disturb other crucial pathways, and determining the optimal timing for intervention. Moreover, while its pathological activation post-MI is detrimental, Wnt signaling is crucial for cardiac health under physiological conditions. Striking a balance between these contrasting roles is paramount for therapeutic success.

### Therapeutic potential of targeting the Wnt/YAP signaling axis in the heart

Given the central role of the Wnt/YAP signaling axis in the progression of cardiac diseases, it represents a promising target for novel therapeutic strategies. However, the development of targeted interventions requires a nuanced understanding of the distinct roles of canonical and non-canonical Wnt signaling, as well as the interaction between Wnt signaling and other pathways implicated in heart failure (Baurand *et al*. 2007; Zelarayán *et al*. 2008).

Preclinical studies have shown that pharmacological inhibitors of Wnt signaling attenuate cardiac hypertrophy and fibrosis, thereby improving cardiac function in animal models of pressure overload–induced heart failure. However, caution must be exercised when developing therapeutic strategies targeting the Wnt/YAP signaling axis, given its pleiotropic effects and the potential for off-target effects (Panciera *et al*. 2017). Moreover, the timing of intervention is crucial, as Wnt signaling has distinct roles at different stages of cardiac remodeling and heart failure progression (Byun *et al*. 2019). Future studies should focus on developing targeted, stage-specific interventions that modulate Wnt/YAP signaling to maximize therapeutic efficacy while minimizing potential adverse effects.

## Conclusions and future perspectives

The Wnt signaling pathway and its interaction with the YAP transcriptional co-activator have emerged as crucial regulators of cardiac remodeling, mechanotransduction, and heart failure progression. However, several critical questions remain unanswered. First, it is not clear how the balance between canonical and non-canonical Wnt signaling is maintained in the heart and how this balance is altered during MI and heart failure. Second, the precise mechanisms by which Wnt/YAP signaling axis affects cardiac fibrosis, angiogenesis, and inflammation are not entirely understood. Third, while studies have shown the potential therapeutic benefits of targeting Wnt/YAP signaling axis, it is essential to consider the stage of heart failure, as Wnt signaling has different roles in the early and late stages of cardiac remodeling. Also, the specific roles of different Wnt ligands and their receptors, as well as the interplay between Wnt signaling and other pathways implicated in heart failure, warrant further investigation.

The exploration of the Wnt signaling pathway in the context of cardiovascular disease and heart failure provides an exciting and promising direction for future research. Unlocking the secrets of the Wnt pathway and its role in the adult heart could open new avenues for therapeutic intervention, and offer hope for millions of patients worldwide afflicted by the devastating effects of cardiovascular disease.
